# Deep Learning‐Assisted Quantification of Atomic Dopants and Defects in 2D Materials

**DOI:** 10.1002/advs.202101099

**Published:** 2021-06-03

**Authors:** Sang‐Hyeok Yang, Wooseon Choi, Byeong Wook Cho, Frederick Osei‐Tutu Agyapong‐Fordjour, Sehwan Park, Seok Joon Yun, Hyung‐Jin Kim, Young‐Kyu Han, Young Hee Lee, Ki Kang Kim, Young‐Min Kim

**Affiliations:** ^1^ Department of Energy Science Sungkyunkwan University (SKKU) Suwon 16419 Republic of Korea; ^2^ Center for Integrated Nanostructure Physics Institute for Basic Science (IBS) Suwon 16419 Republic of Korea; ^3^ Department of Energy and Materials Engineering Dongguk University Seoul 04620 Republic of Korea

**Keywords:** deep learning, dynamic STEM analysis, point defects, scanning transmission electron microscopy, 2D transition metal dichalcogenides

## Abstract

Atomic dopants and defects play a crucial role in creating new functionalities in 2D transition metal dichalcogenides (2D TMDs). Therefore, atomic‐scale identification and their quantification warrant precise engineering that widens their application to many fields, ranging from development of optoelectronic devices to magnetic semiconductors. Scanning transmission electron microscopy with a sub‐Å probe has provided a facile way to observe local dopants and defects in 2D TMDs. However, manual data analytics of experimental images is a time‐consuming task, and often requires subjective decisions to interpret observed signals. Therefore, an approach is required to automate the detection and classification of dopants and defects. In this study, based on a deep learning algorithm, fully convolutional neural network that shows a superior ability of image segmentation, an efficient and automated method for reliable quantification of dopants and defects in TMDs is proposed with single‐atom precision. The approach demonstrates that atomic dopants and defects are precisely mapped with a detection limit of ≈1 × 10^12^ cm^−2^, and with a measurement accuracy of ≈98% for most atomic sites. Furthermore, this methodology is applicable to large volume of image data to extract atomic site‐specific information, thus providing insights into the formation mechanisms of various defects under stimuli.

## Graphical abstract

The deep learning‐assisted quantification algorithm reduces heavy load of data processing for researchers, which has hindered the pace of design and development of 2D transition metal dichalcogenides (2D TMDs). Furthermore, an integrated understanding of the atomic‐scale behavior of point defects in 2D TMDs under environmental stimuli is now available without data reduction or sampling.

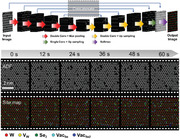

## 1 Introduction

Structural defects and foreign atoms can profoundly affect the functionalities of 2D transition metal dichalcogenides (2D TMDs). Even a limited number of defects or dopants can significantly change the physical properties of 2D TMDs.^[^ ^1^ ^]^ Therefore, precise control of their distributions and local concentrations is the key to tailor the final properties of the materials. To achieve this, an accurate estimation of the type and concentration of the defects and dopants is a prerequisite condition. In this respect, scanning transmission electron microscopy (STEM) has become an indispensable tool for obtaining atomic site‐specific information of nanomaterials because it provides a sufficient resolution for atomic‐scale images with picometer‐level precision.^[^ ^2^ ^]^ In the annular dark‐field (ADF) imaging mode of STEM, where contrast shows atomic number dependency, the atomic sites and the related structural parameters can be quantified through statistical image analysis techniques.^[^ ^3^, ^4^ ^]^ However, it usually demands tedious manual efforts for image processing and quantification.^[^ ^5^ ^]^ Computation codes, which are generally based on motif‐based methods, are used for measuring the positions and intensities of atoms and defects in a STEM image. Although such methods are well developed, they are not optimized sufficiently for efficient data treatment, and they require time‐consuming tasks, even for skillful researchers.^[^ ^6^ ^]^ Furthermore, subjective decisions to interpret the observed signals can be often inevitable if the classification of contrast (or intensity) levels in the STEM image is ambiguous. To solve this issue and to keep in pace with the rapid development, an automated method for the efficient and reliable quantification of defects and dopants needs to be established.

The tradeoff relationship between electron beam irradiation damage and signal‐to‐noise ratio (SNR) is another issue that needs to be considered.^[^ ^7^, ^8^, ^9^ ^]^ The electron dose can be reduced by increasing the scanning rate of the electron probe to mitigate the radiation‐induced damage of the sample. However, ADF images acquired in low electron dose conditions are often too noisy to be analyzed for quantitative estimation of atomic defects. On the contrary, high‐SNR ADF images acquired at high electron doses inevitably suffer from artificial image distortion and/or specimen damage, which ends up garnering invalid information.^[^ ^10^ ^]^ To tackle such a complex situation in the study of radiation‐sensitive materials such as 2D TMDs, a high‐speed recording is recommended as an experimental setup, and the development of a reliable denoising process is required for the accurate estimation of atomic configuration in the materials.

Given the conflicting requisite conditions, establishing a deep learning‐based algorithm for noise reduction and automatic classification can provide a solution for the reliable quantification of atomic defects and dopants in 2D TMDs. Recently, machine learning has improved the efficiencies of image processing techniques, such as image classification and solving inverse problem.^[^ ^11^ ^]^ In particular, with the development of graphical processing units (GPUs), deep learning using artificial neural networks has demonstrated excellent performance in image classification, possibly surpassing experts, in various fields ranging from 3D chemical analysis to autonomous driving to medicine.^[^ ^12^ ^]^ In addition, deep learning has shown exceptional performance in the restoration of images from raw data containing a high level of statistical noise or blurring.^[^ ^13^ ^]^ The advantages of deep learning in the image processing have been recently exploited for analyzing atomic resolution STEM images in terms of automated classification of atomic features, counting the number of defects, and measuring local lattice strains.^[^ ^14^ ^]^ Artificial intelligence‐assisted methods have improved the efficiency of image processing and statistical interpretation of STEM data. However, the accuracy and precision of the results are not comparable to those of professional researchers. Furthermore, there has been no unified solution for image enhancement and automated quantification of the structural features of radiation‐sensitive materials that need to be observed at low dose conditions.

In this study, we propose a new deep learning‐based approach to automatically solve the problems associated with radiation‐sensitive materials regarding their structural chemistry of 2D TMDs. Two essential components were combined in the developed algorithm: image restoration for the denoising process and image classification for quantitative analysis. As a pre‐treatment step for quantitative analysis, an image restoration algorithm, based on convolutional neural networks (CNN), was employed to reduce the statistical noise and enhance the contrast of STEM images. We also developed an automated methodology for reliable quantification of dopants and defects in TMDs with single‐atom precision. The method is based on a deep learning algorithm, i.e., fully convolutional network (FCN), which shows a superior ability in the segmentation of image features.^[^ ^16^ ^]^ By applying our approach to some typical 2D TMDs, i.e., WSe_2_ and MoS_2_ monolayers doped with V (referred hereafter as V‐WSe_2_ and V‐MoS_2_, respectively), we demonstrate that atomic dopants and defects can be precisely mapped with an uncertainty of less than ±0.2%, and can be reliably classified into well‐defined types that are predicted from atomic models. The detection limit of point defects in our approach is estimated to be ≈1 × 10^12^ cm^−2^ while maintaining the requisite atomic resolution (see Note S1 and Figure S1 in the Supporting Information). This high efficiency of the quantification process is also demonstrated by its application to large volumes of data of serial STEM images, which could create a new way of investigating the true atomic structures of radiation‐sensitive materials or the kinetic structural reaction of functional materials under various stimuli, which have remained inaccessible using conventional STEM approaches.

## 2 Results and Discussion

To reduce raster distortion due to the nature of scanning electron beam in atomic‐resolution STEM imaging, which typically impairs the veracity of structural measurements such as lattice strains or atomic positions, we need to acquire ADF STEM images at a high scanning rate. In this study, we have acquired all the ADF STEM images with a fast dwell time of 1 µs pix^−1^ for a 1024 × 1024 pixel resolution, which translates to a recording speed of ≈1 s per frame. At this high‐speed ADF imaging, a high level of statistical noise floor in the image is inevitable, which seriously hinders the reliable structural investigation, even though the artificial scanning distortion can be mitigated. To reduce the noise signals, image filtering based on fast Fourier transform has been widely used. However, this method uses a periodical phase template, which causes an artificial increase of intensity even in the vacant sites, thereby making it unreliable to quantify defects from the filtered image (see Figure S2 in the Supporting Information). Therefore, a trustworthy post‐denoising process after the high‐speed recording of the ADF STEM image needs to be established as a prerequisite for an accurate estimation of atomic defects in 2D TMDs.

To this end, we developed a CNN‐based denoiser algorithm for 2D TMD structure images, which is remarkably effective not only to remove unwanted noise floors but also to enhance the atomic contrast in the ADF image without any artificial generation of signals. In the denoiser algorithm, we used a dilated convolutional kernel, which is different from the conventional CNN models.^[^ ^17^ ^]^ To extract contextual information for image restoration through deep learning, the capacity of the CNN model needs to be significantly large to increase the receptive field of the model, which is achieved using two approaches. One is by increasing the size of the kernel, and the other is by enlarging the network depth. However, such approaches tend to excessively increase the number of parameters considered for the model, thus increasing the risk of overfitting accompanied by large computational costs. As an alternative to this problem, the use of dilated convolutional kernel has been suggested. In a 3 × 3 kernel of *s*‐dilation factors, the kernel size increases by (2*s* + 1) × (2*s* + 1), but all elements of the kernel except for the fixed nine entries, are set to zero. In other words, using a dilated convolutional kernel has the advantage of increasing the capacity of the model without increasing the computational cost.^[^ ^18^ ^]^

**Figure 1:**
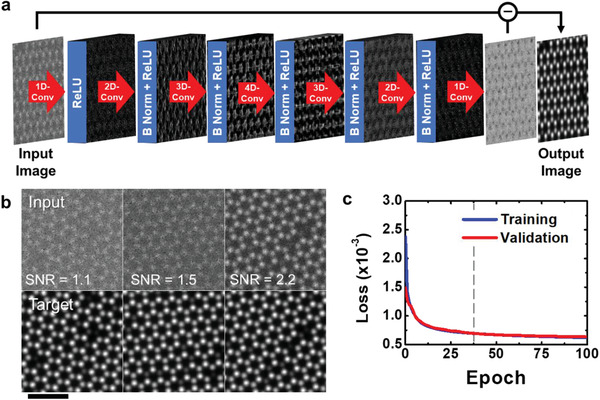
Deep learning‐based denoiser algorithm. a) Deep neural network model constructed for denoising process for ADF STEM image of V‐WSe_2_. b) Examples of training dataset prepared for deep learning of our denoising model. c) Loss graph of the denoising model with the batch size of 32 as a function of evaluation epoch. Scale bar is 1 nm.

Taking advantage of the above‐mentioned approach, we designed a deep learning‐assisted denoising model, as shown in **Figure** **1**a. Our objective was to extract the statistical noise background as an independent signal feature from the experimental ADF image, and subsequently subtract it from the input image to restore the true atomic contrast of the image. To train the model using supervised learning, the training dataset for feature labeling was prepared by ADF image simulations generated by multislice calculations performed using the QSTEM software package.^[^ ^19^ ^]^ The statistical noise signals with Poisson distribution were intentionally added to emulate the experimental ADF STEM images. To apply our denoiser algorithm to the images with different levels of noise background and geometrical rotations, the amount of background noise was adjusted to obtain signal‐to‐noise ratios (SNRs) ranging from 1.1 to 2.2, and the angle of image rotation was arbitrarily selected for the preparation of the simulated images, which were used as the input data in the training session. The lowest allowable SNR (1.1) was determined by considering the consdition that the restoration accuracy can maintain above ≈98% after surveying the effect of SNR with respect to the accuracy (see Note S2 and Figure S3 in the Supporting Information).

Applying it to test STEM images after model training (examples of training dataset prepared for our denoising model is shown in the top row of Figure 1b), we noticed that the unwanted noise floor could be remarkably eliminated, thereby significantly enhancing the atomic contrast of the image. It is apparent that this denoising process improves the accuracy of the classification and the labeling of atomic sites for the subsequent quantification processes. To validate how much training is required to reach a level where both training and test performances remain equivalent within a difference of 5%, we tracked the loss values of the training and test datasets as a function of the training epoch so that the weights can be regularly updated to reduce the loss as the number of evaluations increases (Figure 1c). As a result, it was confirmed that the error in both the training and test sessions of our model can be maintained at a level of 7 × 10^−4^ after 35 epochs without overfitting, and thus we have demonstrated that it is a highly reliable algorithm.

**Figure 2:**
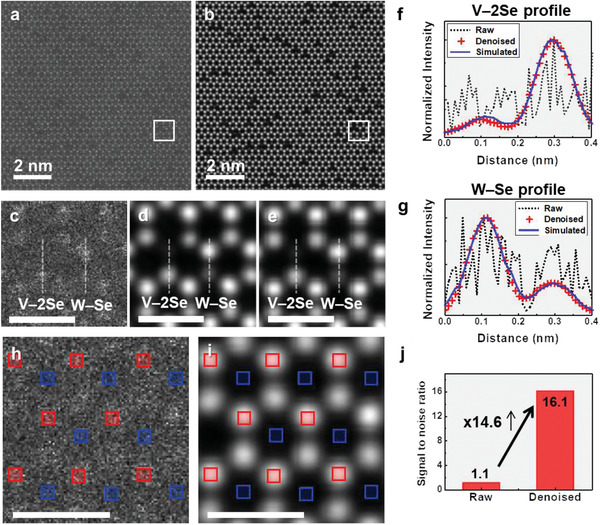
Restoration of noisy experimental ADF‐STEM image of V‐doped WSe_2_ by denoising process. a) A raw ADF STEM image recorded at high scanning rate of 1 µs pix^−1^. b) Restored ADF STEM image after denoising process. c,d) Comparison of the raw and the processed images for the area marked in white box in (a) and (b), respectively. e) Simulated ADF STEM image corresponding to the experimental observation. f,g) Comparison of the vertical intensity profiles of the V‐2Se and W‐Se atomic arrays denoted by white dotted lines in each image. Profiles extracted from raw, denoised, and simulated images are drawn by black dotted, red cross, and blue solid lines, respectively. h–j) Comparison of signal‐to‐noise ratios (SNRs) between the raw and the denoised ADF STEM images: Red and blue boxes marked in the two images, (h) and (j), indicate the sampling areas to estimate the signal intensity of W atom and the standard deviation of noise fluctuation in vacuum space, respectively. The estimated SNRs for the two images are shown in (j). Scale bars are 500 pm.

**Figure** **2**a,b shows how dramatically our denoiser model removes the noise background and improves the atomic contrast of the experimental ADF STEM image of V‐WSe_2_, which was recorded at a high scanning rate. It is worth noting that our denoiser model was proved to be valid for the ADF STEM images of V‐WSe_2_ with a SNR of larger than 1.1 (Figure S4, Supporting Information). Figure 2c,d displays the enlarged structural images before and after the application of the denoising process in the marked regions of Figure 1a,b, which clearly evince that the dominant noise in the raw ADF STEM image was effectively removed. We even noticed that the quality of the processed image is comparable to that of the simulated ADF image (Figure 2e) generated from the atomic model corresponding to the experimental observation. To demonstrate the improvement in atomic contrast, we compared the intensity profiles (Figure 2f,g) extracted from the two different atomic arrays of V‐2Se and W‐Se, which are marked by the vertical dotted lines in Figure 2c–e. Since the intensity in the ADF imaging strongly shows the atomic number dependence if the crystal thickness is uniform,^[^ ^20^, ^21^ ^]^ W and 2Se atoms should exhibit stronger intensity than V or single Se. This contrast characteristic of the atom profiles is clearly observed in the denoised ADF image, whereas the accurate identification of atom positions is seldom possible in the profiles obtained from the raw image because of the significant fluctuations in intensities. Remarkably, we observe that the intensity profiles of atoms in the denoised image are in excellent agreement with those obtained from the simulation. This suggests that the direct quantification of atoms and defects in the denoised ADF STEM image of the V‐WSe_2_ monolayer can be conducted reliably with the optimized deep learning model trained with the simulated ADF image dataset. Our denoiser algorithm, demonstrating the powerful performance of noise reduction, was evaluated to improve the SNR by ≈14.6 times compared to the unprocessed image (see Figure 2h–j).

**Figure 3:**
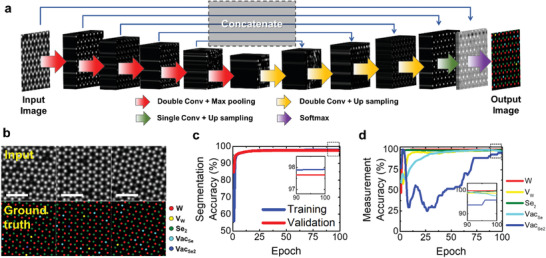
Deep learning‐based algorithm for atomic site classification. a) Deep neural networks model constructed for quantification analysis of ADF‐STEM image of V‐WSe_2_. b) Examples of training dataset for deep learning of our atom segmentation model. c) Pixel‐level accuracy graph of the atom segmentation model as a function of training epoch with a batch size of 32. d) Measurement accuracy graph of the segmentation model compared with human‐based measurements. Scale bars are 1 nm.

The quantification of atomic dopants and defects from atomic‐resolution ADF STEM images by conventional digital processing usually takes approximately one hour for a skilled researcher to obtain a reliable result, which is definitely a labor‐intensive task in cases when there are many images to be analyzed. On this account, a large volume of data obtained from temporally‐resolved sequential imaging process for dynamic studies cannot be completely processed manually. Furthermore, subjective decisions on the classification of weak signals in noisy images have often been made, which unavoidably lowers the veracity of the measurement. Therefore, a reliable automation procedure for quantitative analysis and effective denoising is required. To build a deep learning model that could solve this issue, we adopted the U‐Net model based on the FCN algorithm to construct an automatic flow for the quantification of atomic sites from ADF STEM images in combination with the denoiser algorithm (**Figure** **3**a). Since the FCN model does not use a fully connected layer in the classification model, the location information on the segmentation target is preserved, and variable input size can be applied in the proposed model.^[^ ^16^ ^]^ This is a favorable trait for the quantitative analysis of atomic configuration in STEM images.

However, there are some limitations in the use of a general FCN algorithm, which needs to be considered. First, the learning speed of the model is relatively slow because the existing FCN model learns in the form of a window slide. This characteristic gives rise to an increase in the redundancy of the output data, thus requiring a high cost of GPU memory. Second, there is a trade‐off relationship between the localization and the grasping context of the image when the input image is divided into patch units. If the patch size is set to be large, it is difficult to include the local information, while precisely reflecting the image context. On the other hand, if the patch size is too small, it is difficult to extract the representative image features, whereas information on localizations remains well preserved. To utilize the advantages and suppress the disadvantages, the U‐Net model was employed to establish an efficient and reliable atom segmentation model as an alternative to the typical FCN. The U‐Net model uses an overlap‐tile strategy, which makes no overlapping range of output segmented images, consequently reducing the output size and improving the learning efficiency. We also inserted a skip connection layer so that the image features could be reliably extracted even in a small patch size.^[^ ^22^ ^]^ The diagram representing the architecture of our U‐Net model, which was symmetrically configured with the contracting and expanding paths for quantification analysis of ADF‐STEM image is shown in Figure 3a. To train our U‐Net model for feature segmentation in the V‐WSe_2_ monolayer structure, we classified the possible atomic sites based on experimental observations into five different types: tungsten (W, red), vanadium substituting for tungsten (V_W_, yellow), selenium with no vacancy (Se_2_, green), mono‐vacancy of selenium (Vac_Se_, cyan), and di‐vacancy of selenium (Vac_Se2_, blue). The training dataset was prepared by ADF STEM image simulations using a supercell model with a stochastic distribution of V dopants and Se vacancies. After training, it was confirmed that the atomic configuration of dopants and defects in the V‐WSe_2_ structure was site‐specifically mapped with unambiguity, thus providing critical information on local non‐stoichiometry with single‐atom resolution (Figure 3b). This knowledge is crucial for understanding the optical, electronic, and magnetic properties of this material owing to its strong coupling with the local structures of the dopant‐defect complex.^[^ ^4^, ^23^ ^]^

In the validation test of the learning, we found that the accuracy of the classification was maintained above 97% without overfitting as ground truth after 25 epochs (Figure 3c), which indicated that the established algorithm worked well, and the necessary training had been reliably carried out. To verify the model's performance, we compared the actual results of our automated algorithm with human‐based measurements by cross‐correlation. Fifteen experimental ADF STEM images of V‐WSe_2_ were tested by our algorithm, and the atomic configuration of dopants and defects were analyzed using the conventional human‐based procedure.^[^ ^3^ ^]^ A comparison of 49524 atoms revealed that the detection accuracy for most sites of W, V_W_, Se_2_, and Vac_Se_ was greater than 98%, which proves the high measurement reliability of our method (Figure 3d). As a caveat, we found that the ambiguity in detecting the invisible di‐vacancies (Vac_Se2_) is slightly larger than that of the atomic sites by 3% or 4% after convergence by 100 epochs, which may require more rigorous optimization and training larger models because the accuracy for the detection of di‐vacancy largely fluctuates under insufficient epochs. Nonetheless, our current quantification algorithm shows ample potential to replace tedious human‐based interpretations of STEM images.

**Figure 4:**
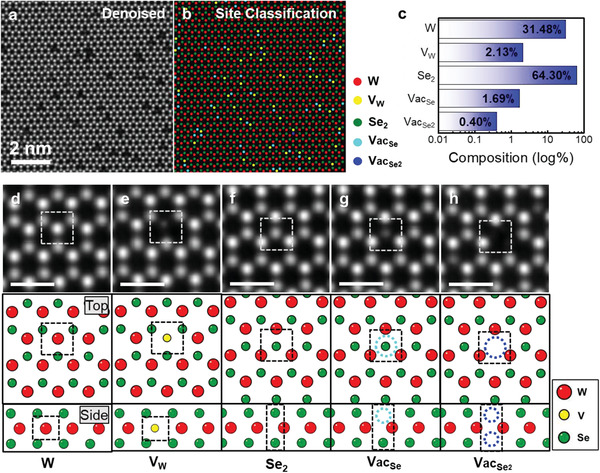
Automated quantification of dopants and defects in V‐WSe_2_ monolayer. a) Denoised ADF STEM image of V‐WSe_2_. b) Deep learning‐assisted atomic site mapping of V‐WSe_2_. c) Result of composition analysis for all atomic sites of ADF STEM image of V‐WSe_2_. d–h) (top panels) The five representative atomic sites from the ADF STEM image identified by the developed algorithm: W, V substituting for W (V_W_), Se_2_, Se mono‐vacancy (Vac_Se_), and Se di‐vacancy (Vac_Se2_). (bottom panels) Atomic models corresponding to the types of the identified sites. Scale bars are 500 pm.

**Figure** **4** displays a representative application of our automated quantification algorithm to the experimental STEM image of the V‐WSe_2_ sample. After denoising (Figure 4a), our quantification model demonstrates that automated classification of atomic sites in the sample can be precisely performed in seconds (Figure 4b). The results of the site quantification revealed that the contents of the total chalcogen vacancy (Vac_Se_ + Vac_Se2_) and the V dopant in the sample were 2.09% and 2.13%, respectively (Figure 4c), which is consistent with a previous report based on human‐performed data processing.^[^ ^4^ ^]^ As our U‐Net‐based quantification model was trained, the five different atomic sites including the V dopant and intrinsic point defects, which are energetically formable,^[^ ^24^ ^]^ were well identified and displayed a one‐to‐one correspondence with the known atomic models (Figure 4d–h). Once the deep learning‐assisted quantification process is successfully finished, the geometric configuration of dopants and vacancies over the sample can be further analyzed (Figure S5, Supporting Information) and this kind of routine for the data interpretation can be readily added into the algorithm to obtain a rich information about the local structural behavior of defects in 2D TMDs.^[^ ^23^ ^]^

Since this method is based on the procedure of counting atoms appearing in the ADF STEM image, the determination of the imaging field‐of‐view (FOV) by microscope magnification to constrain the total number of atoms that are being imaged is an important factor that should be considered for estimating the minimum detectability of dopants or defects. In this regard, we simulated ADF images of the V‐WSe_2_ model with a fixed concentration of selenium vacancies at different FOVs and compared the quantification results of the vacancy concentration using our algorithm (see Note S1 and Figure S1 in the Supporting Information). As a result, unless the FOV is smaller than 10^2^ nm^2^ that contains 3150 atoms, a high level of measurement precision (less than ±0.2%) on the vacancy concentration is maintained. In this case, the detection limit of point defect by our approach was estimated to be ≈1 × 10^12^ cm^−2^. With regard to the efficiency, our algorithm requires 3 s per 1024 × 1024 pixel STEM image in quantification by our GPU‐based calculation, whereas a skillful researcher usually requires approximately 1 h for the same task, including data visualization. This indicates that the established methodology provides quantified results with a high efficiency of ≈1200 times faster than the currently used data processing techniques. More importantly, our results suggest that the deep learning‐supported analysis using high‐speed STEM imaging may open a new way towards low‐dose electron experiments for radiation‐sensitive nanomaterials as well as real‐time structural investigations of materials under various external stimuli because it is efficient to process massive giga‐scale datasets, which is beyond the capability of human beings.

**Figure 5:**
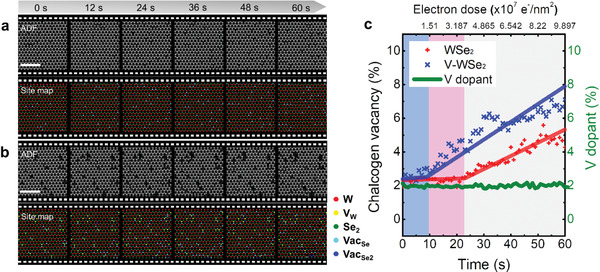
Application of the automated quantification algorithm to large imaging data volumes of (doped) WSe_2_ showing dynamic defect evolutions under electron beam irradiation. a,b) (top) Series of ADF STEM images of pristine WSe_2_ and V‐WSe_2_ sequentially recorded under electron beam irradiation and (bottom) corresponding atom‐site classification maps obtained from the established deep learning algorithm. Note that the ADF images shown here are the ones that were denoised by the denoiser algorithm. c) Plot of variations in concentrations of Se vacancies and V dopants in the WSe_2_ and V‐WSe_2_ as a function of electron beam irradiation time (or total electron dose). Note that color‐shaded regions in the graph indicate the threshold times (or doses) allowed to capture the intact structures of WSe_2_ (blue) and V‐WSe_2_ (pink) monolayers, respectively, without radiation‐induced structural damage. Scale bars in the ADF images are 2 nm.

To demonstrate the promising applicability of our quantification algorithm in the dynamic study of structural evolution under environmental stimuli, we surveyed the electron beam‐induced changes in pristine (WSe_2_ and MoS_2_) and V‐doped (V‐WSe_2_ and V‐MoS_2_) samples as a function of electron dose under a fixed dose rate of ≈1.7 × 10^6^ e^−^ s^−1^·nm^−2^. A 3D image dataset built by stacking multiple images sequentially recorded at a high acquisition rate of 1 µs pix^−1^, which was translated to an acquisition time of ≈1 s for a 1024 × 1024‐pixel image, was applied to our quantification algorithm to explore the dynamics of vacancy in response to electron beam stimulus (**Figure** **5**). Continuous electron bombardment at high accelerating voltages usually induces structural modifications by elastic knock‐on collisions, breakage of ionic bonds by inelastic radiolysis reaction, or other phenomena such as heating and electrostatic interactions.^[^ ^8^, ^9^, ^25^, ^26^, ^27^, ^28^, ^29^ ^]^ In 2D TMDs under a relatively low accelerating voltage, the radiolysis interaction between electrons of sample atoms and electron beam occurs dominantly, preferentially accompanying the sublimation of chalcogen ions.^[^ ^3^, ^25^ ^]^ This radiation‐induced change is known to appear as a threshold phenomenon depending on the total electron dose, and can be used as a means of point defect engineering and for fundamental studies of structural response under harsh irradiation environments.^[^ ^26^ ^]^ Thus, quantitative monitoring of electron beam‐induced structural behavior in 2D TMDs is crucial for precise engineering of point defects and structural modifications at an atomic‐level, and for establishing safe conditions to obtain valid information of intact structures observed under STEM. To this end, sequential acquisition of atomic‐resolution STEM images is required to resolve the temporal changes in structure.

A series of ADF STEM images of WSe_2_ and V‐WSe_2_, acquired under electron beam irradiation and at a high acquisition rate, are shown in Figure 5a,b, respectively. The initial contents of both chalcogen vacancy and V dopant in the two samples were estimated to be ≈2%, as shown in Figure 4 (Table S1, Supporting Information). Several ADF STEM images and the resulting site maps automatically obtained by our deep learning algorithm were chosen for display. Even though the raw ADF images recorded at fast acquisition rate (1 µs pix^−1^, SNR = 1.1) in this experimental setup were quite noisy, the denoised ADF images showed a remarkable quality with a highly improved SNR (SNR = 16.1), thus facilitating a reliable site classification. Full‐size images and the corresponding atom site maps are given in Movies M1 and M2 in the Supporting Information, respectively, which were cropped and enlarged for better visualization in Figure 5. The application of the automated quantification algorithm revealed that a large volume of the raw data (stacked by 60 ADF images) can be efficiently interpreted within less than 3 min, which may require approximately 60 h in case a human works at a constant speed. Additionally, the quantification results provide integrated physical information on the spatial distribution of defects and dopants, and their temporal changes under electron beam irradiation, as shown in Figure 5c. We observed a typical dose‐dependent phenomenon of electron beam‐induced generation of chalcogen vacancies for the two samples. However, the tolerance to electron beam irradiation was found to be quite different between the two samples. The V‐doped WSe_2_ was approximately twice as sensitive to electron dosage as compared to the pristine WSe_2_; Se vacancies in the V‐doped WSe_2_ started to rapidly increase after the electron dose of ≈1.5 × 10^7^ e^−^ nm^−2^ (blue graph in Figure 5c), whereas it began to increase after the electron dose of ≈3.5 × 10^7^ e^−^ nm^−2^ (red graph in Figure 5c) in pristine WSe_2_. Intriguingly, the content of V dopants under continuous electron beam irradiation remained almost constant (green graph in Figure 5c), which suggests that anions are more susceptible to the radiolysis interaction with the electron beam than cations, which is in accordance with many studies on electron beam interactions.^[^ ^3^, ^9^, ^21^, ^27^, ^30^ ^]^ This dynamics of defect generation behavior was first revealed from the massive image dataset with the help of deep learning‐assisted quantification, which does not require data sampling to reduce the load of interpretation at the cost of accuracy.

The higher susceptibility of the doped WSe_2_ to the electron beam irradiation could be attributed to local changes in the bonding environments due to doping. To understand this characteristic difference, we compared the formation energies of chalcogen defects according to their probable spatial configurations in the framework of density functional theory (DFT) calculations (Figure S6, Supporting Information). The results show that the energies required for the formation of all configurations in the two samples were similar. The relatively stronger binding energies for the dopant‐vacancy complexes are analogous to Cr doped WSe_2._^[^ ^31^ ^]^ Given that the initial content of chalcogen vacancies was almost the same in the two samples (Table S1, Supporting Information), it is expected that the electron beam‐induced chalcogen vacancies were triggered at an earlier stage of electron beam irradiation in V‐WSe_2_, as shown in Figure 5. This is because the complexes such as V–third neighboring Vac_Se_ and V–two Vac_Se_ as first neighbors have slightly lower binding energies compared to the vacancy configurations with W in pristine WSe_2_. It is worth noting that the elemental loss of W atoms and V dopants as well as that of Se atoms in the V‐doped WSe_2_ began after 67 s of electron beam irradiation (not shown here), which is considered the maximum endurance of the material to the electron beam irradiation. Further irradiation caused collapse or destruction of the material structure, accompanied by the formation of growing holes due to the accelerated loss of elements, which is beyond the scope of point defect engineering.

Extending the applicability of our automated algorithm to other 2D TMDs, the dynamic defect evolution of pristine MoS_2_ and V‐doped MoS_2_ under electron beam irradiation was efficiently examined and quantitatively compared, as shown in Figure S7 in the Supporting Information. The full‐size images and the corresponding atom site maps of the undoped MoS_2_ and V‐MoS_2_ are given in Movies M3 and M4 in the Supporting Information, respectively. The tolerance to elastic bombardment by incident electron beam tends to be higher in selenides than in sulfides, because the activation energies of chalcogen sputtering and displacement are relatively higher for larger atomic numbers.^[^ ^28^ ^]^ Furthermore, anion (sulfur) vacancies can be more readily formed in sulfides because of their higher sensitivity at a fixed electron dose as a result of larger inelastic interactions with incident electrons.^[^ ^30^ ^]^ Therefore, we expect that 2D MoS_2_ would react more readily to electron irradiation to form point defects therein. Indeed, it is revealed that the threshold electron dose (≈0.8 × 10^7^ e^−^ nm^−2^) required for the formation of radiation‐induced defects in MoS_2_ is much smaller than that in WSe_2_; the point defects start to form after about 5 s under the same condition of the electron beam irradiation, which is about two times smaller than that of WSe_2_. Interestingly, we found that there was no notable difference between the undoped MoS_2_ and V‐MoS_2_ in the generation of radiation‐induced defects, except for a small deviation in the initial chalcogen vacancy content between them, even though the formation energies of sulfur vacancies near the V dopants are lower than those for the equivalent structural configurations in the pristine MoS_2_. In the V‐MoS_2_ sample, our quantification algorithm estimated the concentration of V dopant to be less than 0.4%, which is much smaller than that of intrinsic chalcogen vacancies (≈1.73%) (Table S1, Supporting Information). Therefore, the contribution of the V dopants to the electron beam irradiation effect, which creates extrinsic point defects, would be insignificant as compared to the contribution of intrinsic sulfur vacancies at high concentration.

The exemplar applications of our deep learning algorithm to the (doped) WSe_2_ and MoS_2_ samples have shown that more precise engineering of point defects is possible to tailor the physical properties of 2D TMDs under electron beam irradiation. Once the deep learning‐assisted quantification is successfully performed, a more useful statistical analysis for tracking dynamic trajectories of point defects in spatiotemporal dimension can be conducted (Figure S8 in the Supporting Information) as demonstrated by Maksov et al.,^[^ ^15^ ^]^ which can yield a wealth of information for understanding the defect evolution and transition under electron beam stimulus. It should be noted that the specific values of the electron dose to trigger the generation of point defects in 2D TMDs might be different depending on the synthesis method, sample preparation, and microscope parameter setup. These extrinsic factors, which should be considered in individual cases, can further justify the need for systematic investigation using an automated quantification algorithm. From our automated analytics, we can deduce what physically happens in the sample subjected to electron beam irradiation on an atomic scale. This is important for not only developing a fundamental understanding of the electron beam–sample interaction but also for precisely defining the critical dose condition that triggers structural changes in the materials at the atomic‐scale.

## 3 Conclusion

To summarize, we developed a deep learning‐assisted algorithm for the efficient classification and quantification of atomic dopants and defects in 2D TMDs. To demonstrate the perfoemance of the algorithm, a variety of defects and dopants in quintessential 2D TMD monolayers and their doped forms (WSe_2_, MoS_2_, V‐doped WSe_2_, and V‐doped MoS_2_), which are emerging as promising 2D magnetic semiconductors, was efficiently quantified with high accuracy; the accuracies were comparable to that of human‐based data interpretation. Our deep learning algorithm showed an exceptionally high efficiency for site classification on an atomic‐resolution image dataset—1200 times faster than the currently popular analysis technique performed by human. Our approach also demonstrated excellent performance in the reduction of statistical noise of STEM images recorded at high speeds and in the handling of a large data volume of serial STEM images, likely extending its application to in situ or low dose STEM studies.

## 4 Experimental Section

4.1

4.1.1

##### Synthesis of Pristine and V‐Doped TMD Monolayers

Pristine and V‐doped WSe_2_ (or MoS_2_) samples were grown using an atmospheric CVD system. The liquid precursor for the growth of WSe_2_ (or MoS_2_) was prepared by mixing ammonium metatungstate, (or ammonium molybdate) and NaOH promoter. Additionally, ammonium metavanadate was introduced into the liquid precursor for V‐doping. The details of which are presented in Ref. [4]. The prepared liquid precursor was subsequently spin‐casted onto a SiO_2_/Si substrate, followed by selenization (or sulfurization) in the CVD chamber. To separately control the evaporation of selenium (or sulfur) and the reaction temperature, a two‐zone furnace CVD was adopted. For the growth of WSe_2_ (or MoS_2_), the Se (or S) was heated up to 400 °C at a rate of 50 °C min^−1^ (or 25 °C min^−1^), while the temperature of the substrate zone was elevated to 760 or 850 °C. Nitrogen and hydrogen gases at flow rates of 500 and 5 sccm, respectively, were injected during the CVD process. After the growth of the TMD, the furnace was naturally cooled to room temperature.

##### DFT Calculations

Spin‐polarized DFT calculations were conducted using the Vienna ab initio simulation package.^[^ ^32^ ^]^ Here, the revised Perdew−Burke−Ernzerhof type exchange and correlation functional was employed.^[^ ^33^ ^]^ The projector augmented wave (PAW) method was used for ion interaction. The Brillouin zone was sampled using a Г‐centered 3 × 3 × 1 k‐point mesh, while the electronic states were smeared using the Methfessel−Paxton scheme with a broadening width of 0.1 eV. The electronic wave functions were expanded in a plane wave basis with a cutoff energy of 400 eV and the atomic relaxation was continued until the Hellmann−Feynman forces acting on the atoms were less than 0.02 eV Å^−1^. All vacancy‐containing structural calculations were performed using MoS_2_, V‐MoS_2_, WSe_2_, and V‐WSe_2_ monolayers considering 5 × 5 supercells of MoS_2_ and WSe_2_, respectively. A vacuum layer thicker than 16 Å was added onto each surface to eliminate the possible interlayer interactions. The vacancy formation energy is defined as *E*_f_ = (*E*_V_ − *E*_0_) + *µ*_i_, where *E*_V_ is the total energy of the vacancy‐containing TMD structure, *E*_0_ is the total energy of the TMD structure before the introduction of vacancy, and *µ*_i_ is the chemical potential of the vacancy atom.

##### STEM Imaging

Atomic‐scale structure imaging of the V‐WSe_2_ monolayer was performed using an aberration‐corrected STEM (ARM200CF, JEOL Ltd.) operating at 80 kV with a probe current of 25 pA. The semi‐convergence angle of the electron probe was 23 mrad and the angle range of the ADF detector was ≈68–280 mrad. The ADF images were recorded at a scanning rate of 1 µs pix^−1^ for a 1024 × 1024‐pixel image, which translates 1.05 s as the acquisition time per frame. Under the probing conditions, the electron dose rate was evaluated to be ≈1.7 × 10^6^ e^−^ s^−1^ nm^−2^. Simulated ADF STEM images were generated using the multislice calculation‐based QSTEM software package with the same microscope parameters used in the experimental observation.^[^ ^19^ ^]^

##### Construction of Neural Network and Deep Learning

The structure of the denoising model consists of seven layers: the first layer was composed of a dilated convolutional block and rectified linear unit (ReLU) block, and the next five layers were a combination of a dilated convolution block, a batch normalization block, and a ReLU block. The last layer was set as a dilated convolution block, and the kernel size of the convolutional block in each layer increases by the dilated factor value starting from the default size of 3 × 3 pixels. The dilated factors for each layer were 1, 2, 3, 4, 3, 2, and 1. The number of kernels in each layer, excluding the first and last layers, was 64.

To train the denoising model, a training dataset was prepared with the simulated ADF images of V‐WSe_2_ having stochastic distributions of V dopants and Se vacancies. The input data were created by adding Poisson noise to the simulation image, and the total simulated images without the noise were used as the source of target data. For data augmentation, multiple 256 × 256‐pixel images were extracted from each 1024 × 1024‐pixel simulated image, and the rotation angle and crop position were randomly set during the extraction process. Among the extracted images, 11520 images and 3840 images were used as the training and validation sets, respectively. With this dataset, the model was trained for up to 100 epochs with a batch size of 32, and it was confirmed from the loss graph that the model was well trained without overfitting.

The atom classification model for the V‐WSe_2_ structure had a U‐Net architecture, which is known to have a high performance in semantic segmentation in various fields.^[^ ^22^, ^34^ ^]^ This model consisted of a contracting path that captures the context of an image, and an expansive path that up‐samples the feature map. The contraction part was composed of five types of layers: the first layer was a double convolutional layer, and the rest were a combination of max‐pooling and double convolutional layers. The number of feature maps increased to 32, 64, 128, 256, and 512 throughout each layer. The expansion part was also composed of five layers, out of which four layers were a combination of double convolution and up‐sampling, and the last layer of the double convolutional layer for atom classification. In particular, the last layer had six feature maps to categorize each pixel into six labels using softmax. Label 0 was the background, and labels 1 to 5 represented W, V_W_, 2Se, Vac_Se_, and Vac_Se2_, respectively. A double convolutional layer is a form in which the order of the 3 × 3 convolutional block, batch normalization block, and ReLU block is repeated twice.

To train the atom classification model, simulated ADF images of V‐WSe_2_ were also used to configure a training dataset. The input dataset consisted of cropping the simulated image to a 256 × 256‐pixel image with random rotation and crop position. The target dataset was reconstructed into a pixel‐labeled image by referring to the input data. From this dataset, 17280 images were used as the training set and 5760 images were used as the validation set. The model was trained for up to 100 epochs with a batch size of 32, and the accuracy for classification on the dataset was confirmed to be > 97% by pixel‐based measurement, but not overfitted.

Python was used as the main programming language in this study. The deep learning algorithm in this study was constructed using PyTorch (version 1.5.1), which is a Python deep‐learning library. All the developed deep learning routines implemented as home‐built open sources are available at https://github.com/SKKU‐STEM/2D_TMD_Quantification_with_Deeplearning. The quantitative analysis was performed with Atomap (version 0.2.1), which was constructed in the Python library for analyzing atomic resolution STEM images.^[^ ^35^ ^]^ Data augmentation for configuring the training dataset was proceeded using HyperSpy (version 1.6.1), which is an open source of Python library that provides tools to facilitate the interactive data analysis of multidimensional datasets.^[^ ^36^ ^]^

## Conflict of Interest

The authors declare no conflict of interest.

## Supporting information

Supporting Information

Supporting Movie 1

Supporting Movie 2

Supporting Movie 3

Supporting Movie 4

## Data Availability

The data that support the findings of this study are available from the corresponding author upon reasonable request.
