# Supplementary material for: Deep Learning‐Assisted Quantification of Atomic Dopants and Defects in 2D Materials
Source: Adv Sci (Weinh). 2021 Jun 3;8(16):2101099. doi: 10.1002/advs.202101099 (PMC8373156; doi:10.1002/advs.202101099)
Supplement: Supplementary file 1 — Supporting Information [file ADVS-8-2101099-s001.pdf]

WILEY-VCH

© 2021 Wiley-VCH GmbH

ADVANCED SCIENCE
Open Access

Supporting Information

for *Adv. Sci.*, DOI: 10.1002/advs.202101099

Deep learning-assisted quantification of atomic dopants and defects in two-dimensional materials

*Sang-Hyeok Yang, Wooseon Choi, Byeong Wook Cho, Frederick Osei-Tutu Agyapong-Fordjour, Sehwan Park, Seok Joon Yun, Hyung-Jin Kim, Young-Kyu Han, Young Hee Lee, Ki Kang Kim, and Young-Min Kim**

Supporting Information

Deep learning-assisted quantification of atomic dopants and defects in two-dimensional materials

*Sang-Hyeok Yang, Wooseon Choi, Byeong Wook Cho, Frederick Osei-Tutu Agyapong-Fordjour, Sehwan Park, Seok Joon Yun, Hyung-Jin Kim, Young-Kyu Han, Young Hee Lee, Ki Kang Kim, and Young-Min Kim**

This supporting information includes:

1. Notes S1 and S2
2. Table S1
3. Figures S1 to S8
4. Movies M1 to M4 (uploaded separately):

   M1. Full-size ADF STEM images and the corresponding atom site maps of WSe_2.

   M2. Full-size ADF STEM images and the corresponding atom site maps of V-WSe_2.

   M3. Full-size ADF STEM images and the corresponding atom site maps of MoS_2.

   M4. Full-size ADF STEM images and the corresponding atom site maps of V-MoS_2.

Note S1. Estimation of measurement precision and minimum detectability

The total number of atoms that can be captured in the image is determined in a fixed field-of-view (FOV) of ADF STEM imaging. As the imaging FOV is decreased (in other words, as the magnification is increased), the number of atoms captured in an image gets reduced. Therefore, the measurement precision for the point defects could become poor at small FOV. In contrast, the analysis of atom position becomes difficult in an excessively large FOV owing to insufficient image resolution. Hence, it is important to track how the measurement error can increase with decreasing FOV to define an allowable measurement precision while maintaining atomic resolution. From the results in Figure S1, we see that the measurement error is substantial for the small FOVs of 10^2 nm^2 for both concentration measurements of V dopant and Se vacancies, even though the averaged concentrations obtained from more than 100 selected images show similar values over the entire FOV range. To secure sufficient image resolution for atom feature segmentation, we chose the imaging FOV of the ADF STEM to be 10^2 nm^2. Under these imaging conditions, the measurement precision and minimum detectability were obtained as ~±0.2% and ~1×10^{12} cm^{-2}, respectively.

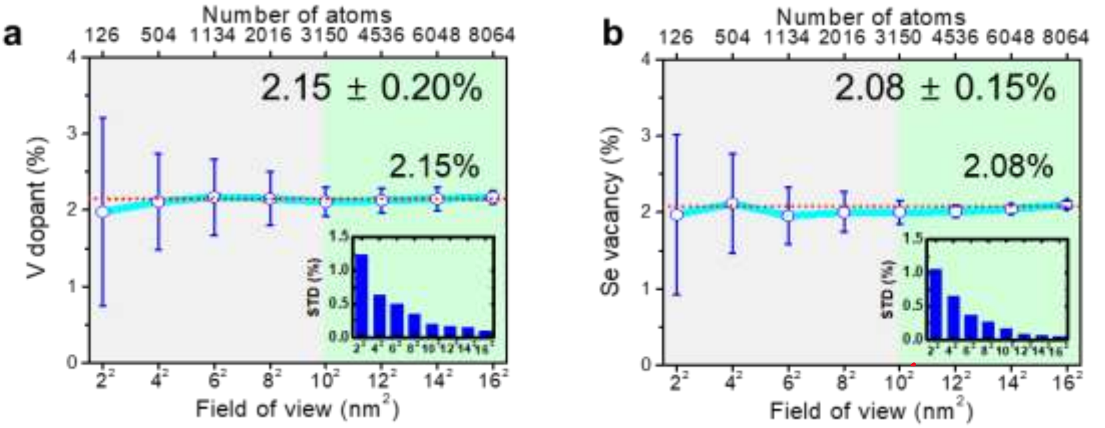

Figure S1. Measurement error and detection limit for the defect quantification as a function of imaging field-of-view (FOV) via atomic-resolution ADF STEM. Measurement errors (standard deviations, insets) for the contents of a) V dopants and b) Se vacancies in WSe_2 monolayer according to the change in the FOV of STEM imaging. To estimate the measurement error of our method, we used simulated ADF STEM images as test data (100 images for every FOV), which were derived from the V-WSe_2 supercell model with the dimension of 20 x 20 nm^2. The contents of V dopants and Se vacancies (Vac_{Se}, Vac_{Se2}) randomly seeded into the supercell structure were 2.15 and 2.08% (marked by red dotted lines), respectively, which were obtained from the experiments. The test images with

different FOVs were arbitrarily selected within the large simulated ADF image for the statistical estimation of the defect concentration.

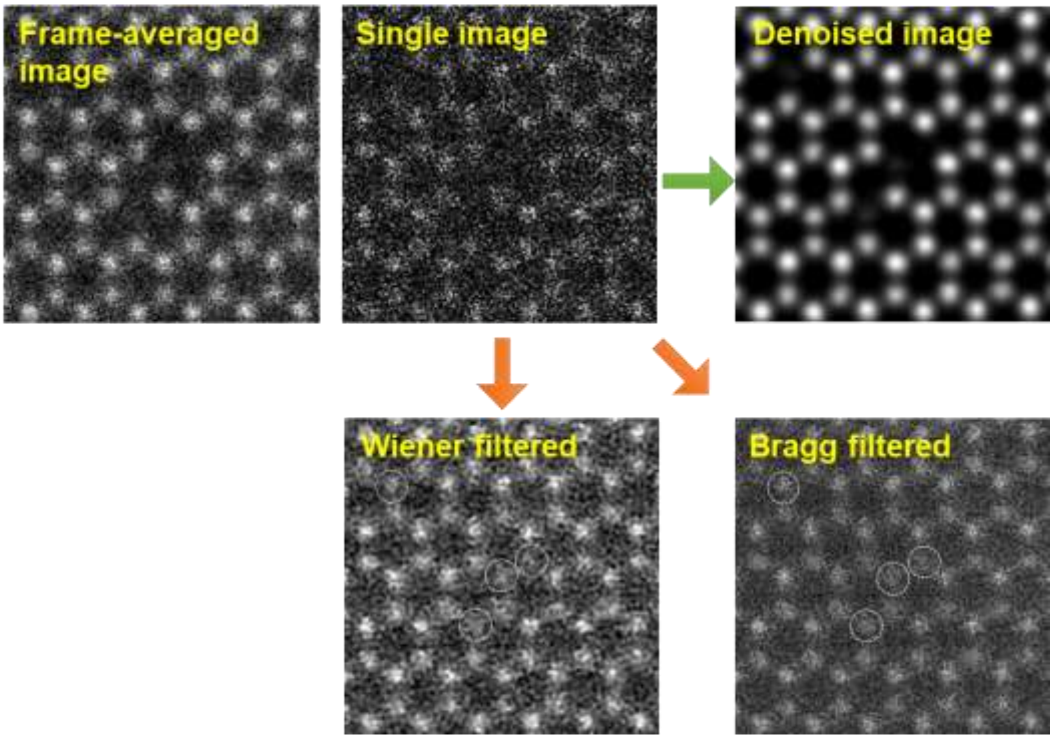

Figure S2. An experimental demonstration of the artificial enhancement of the signal intensity of defects in a low SNR STEM image after the application of fast Fourier transform-based noise filtering process such as Wiener filtering and Bragg pattern filtering. Note that our denoiser model works properly without the false generation of signal intensity and the frame-averaged image with high SNR is provided as a ground truth image for comparison.

Note S2. The effect of signal-to-noise ratio (SNR) with respect to restoration accuracy of the denoising algorithm

At a too low value of SNR, our denoising model would not work properly and artificially change the relative intensities of atoms and defects. Generally, we would expect that as the SNR is gradually decreased, the measurement error becomes notable below a certain value of SNR, which limits the practical application of our deep learning-assisted denoising algorithm. To find the critical SNR, we evaluated restoration accuracy of the site classification as a function of SNR from a series of simulated images generated with different SNRs. As a result, we can see that the restoration accuracy maintains above 98% for all atomic sites and defects of V-doped WSe_2 when the SNR is larger than 1.1. However, statistical error in restoration accuracy is noticeably increased from the images with the values of SNR below 1.1 (Fig. S2a-c). We see that the classification error arises from the fake features generated after the application of denoising algorithm (Fig. S2d-f). Based on the

result, we can define the fastest acquisition rate in STEM imaging for the V-WSe_2 sample, while maintaining a reliable restoration accuracy of above ~98%. To satisfy the condition that the SNR should be higher than 1.1, experimental ADF STEM images of the V-WSe_2 sample were recorded at the high scanning rate of 1 μsec/pix in this study. For V-MoS_2, we found that the minimum limit of SNR should be ~1.5 to maintain the classification accuracy similar to the case of V-WSe_2. This is ascribed to the lower scattering contrast of MoS_2 at the same electron probe condition (see Fig. S2g-i). As in the V-WSe_2, we can see that fake feature contrast appears in the STEM image with the SNR of below 1.5 by the denoising process (Fig. S2j-l). These results suggest that the fastest allowable scanning rate should be determined after carefully surveying the effect of SNR with respect to the restoration accuracy.

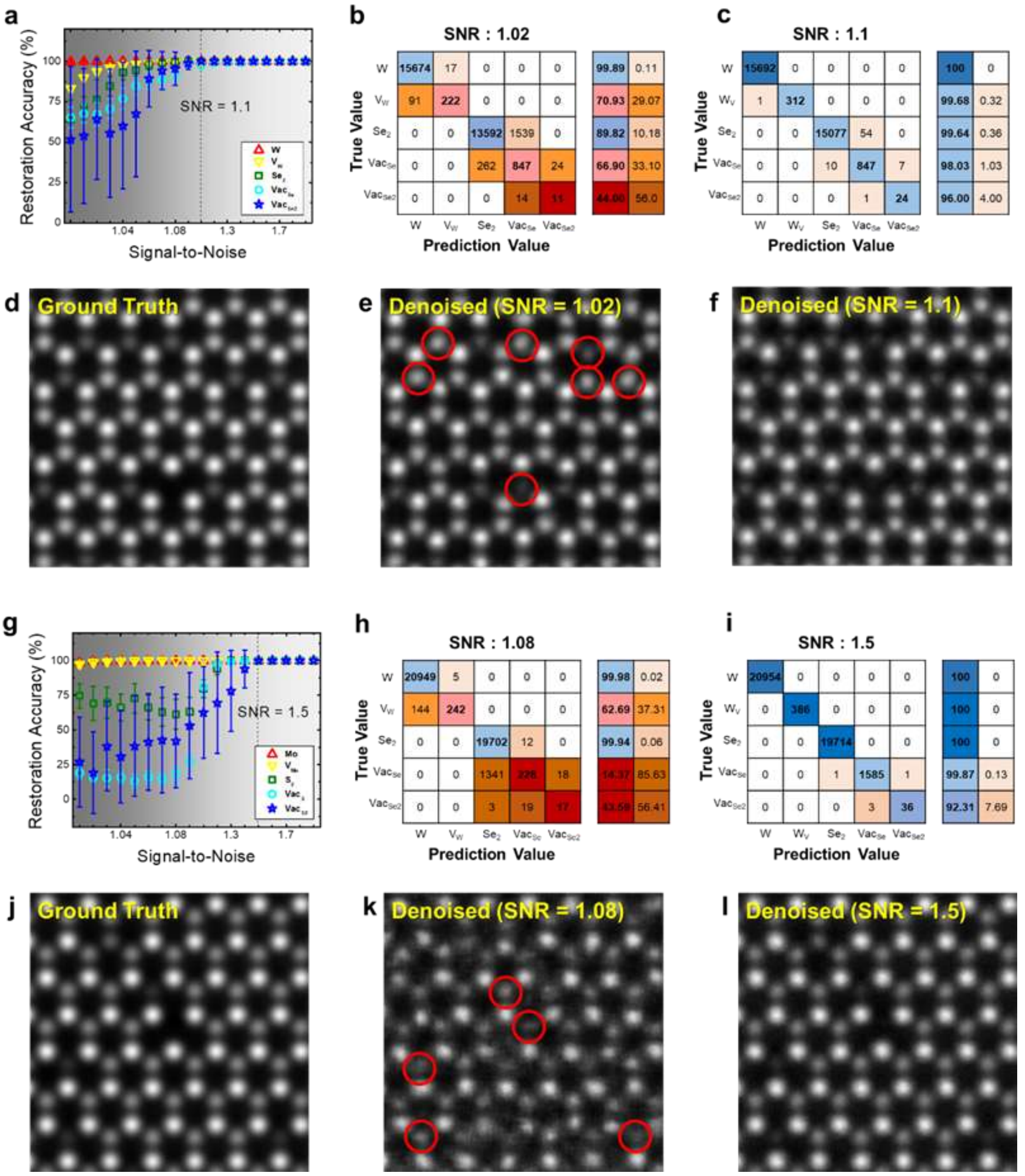

Figure S3. The effect of signal-to-noise ratio (SNR) with respect to restoration accuracies. a,g) Plots showing changes in restoration accuracies of our denoising algorithm for atoms and defects in the respective V-WSe_2 and V-MoS_2 as a function of SNR. b,c,h,i) Confusion matrices displaying the classification results below or at the critical value of SNR for the two respective samples. Note that the critical SNRs at which the restoration accuracy for most atomic sites except for Se di-vacancy maintains under 98% were differently estimated to be 1.1 and 1.5 for the two samples, V-WSe_2 and V-MoS_2, respectively. The diagonal boxes from top-left to bottom-right in the matrices represent the correct matching of site classification to the ground truth. d-f, j-l) Comparison of the ground truth image with the images generated below or at the critical SNR after denoising treatment for the two samples, V-WSe_2 and V-MoS_2, respectively. Note that fake feature contrast appears in the STEM images with low SNR values below the critical SNR after denoising (see red circles in e and k).

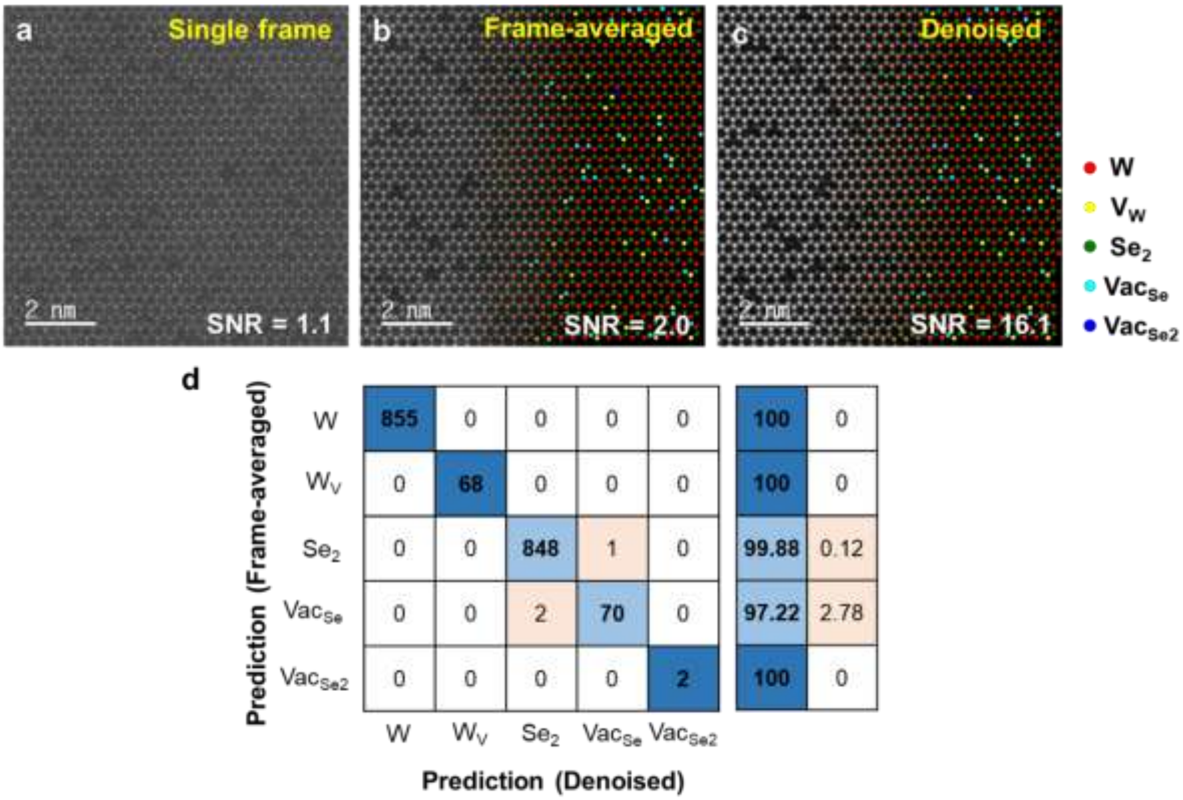

Figure S4. Comparison of the site mapping results of the denoised STEM image with the ground truth STEM image. a) The raw ADF STEM image used for our denoising algorithm shown as Figure 2a, which was extracted from image data volume collected before the onset of electron beam-induced damage. b) A high SNR STEM image obtained by averaging 10 images in the image data volume. c) Restored ADF STEM image after denoising process of a shown as Figure 2b. Note that the right half parts of b and c are the results of site classification to evaluate the consistency between the ground truth and the denoised images. d) Confusion matrix obtained by matching of the site classification results for the frame-averaged and the denoised ADF STEM images.

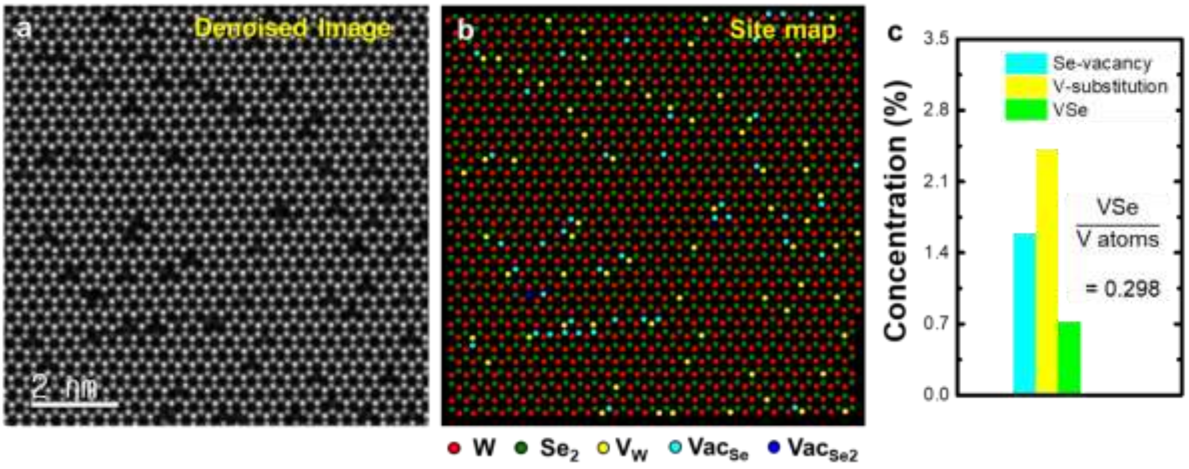

Figure S5. An example of dopant-vacancy pair distribution analysis obtained from the site map of V-WSe_2 resulted from our quantification algorithm. a) Atomic structure of V-WSe_2 denoised from a single ADF STEM image and b) the site map obtained from our deep learning quantification algorithm. c) Statistical analysis of V-WSe_2 for Se vacancies, V substitution, V-Se complexes obtained from the site map.

Table S1. Concentrations of total chalcogen vacancy and V dopant in (doped) WSe_2 and MoS_2 samples measured by the deep learning quantification algorithm developed in this study.

Material	Total chalcogen vacancy (%)	V dopant (%)
WSe_2	1.94	0
V-WSe_2	2.09	2.13
MoS_2	1.54	0
V-MoS_2	1.73	0.38

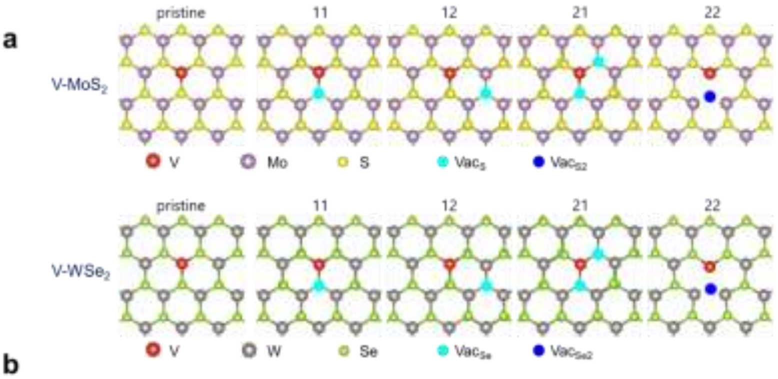

b

Vacancy	Vacancy formation energy (eV)			
	MoS_2	V-MoS_2	WSe_2	V-WSe_2
11	5.85	5.59	5.31	5.32
12	5.85	5.81	5.31	5.29
21	6.31	6.19	5.73	5.70
22	6.32	6.15	5.50	5.57

Figure S6. Formation energies of various V dopant-chalcogen vacancy complexes in the V-WSe_2 and V-MoS_2 monolayers. a) Four possible atomic configurations of V dopant-chalcogen vacancy complexes in the two structures: 11 = V–Vac_{Se} pair as first neighbor, 12 = V–third neighboring Vac_{Se} pair, 21 = V–two Vac_{Se} as first neighbors, 22 = V–Vac_{Se2} pair, respectively. b) DFT-derived formation energies for the four V dopant-chalcogen vacancy complexes in the two structures. Note that atomic configurations of 11 and 12 are equivalent to Vac_{Se} (monovacancy) in the undoped WSe_2 and MoS_2 monolayers, while 21 and 22 denote second neighboring Vac_{Se}–Vac_{Se} pair and Vac_{Se2} (divacancy), respectively.

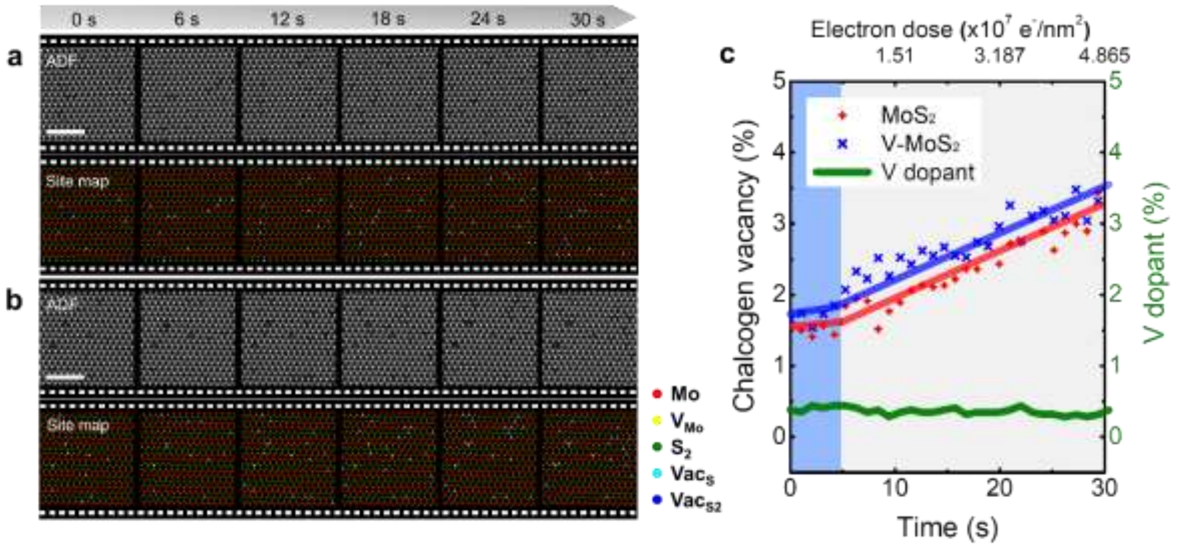

Figure S7. Additional application of the automated quantification algorithm to large imaging data volumes of (doped) MoS_2 showing evolution of dynamic defects under electron beam irradiation. a,b) (top) Series of ADF STEM images of pristine MoS_2 and V-MoS_2 sequentially recorded under electron beam irradiation and (bottom) the corresponding atom-site classification maps obtained from the established deep learning algorithm. Note that the ADF images shown here are the ones that were denoised by the denoiser algorithm of V-MoS_2 version. c) Plot of variations in concentrations of S vacancies and V dopants in the MoS_2 and V-MoS_2 as a function of electron beam irradiation time (or total electron dose). Note that the color-shaded regions in the graph indicate the threshold times (or doses) allowed to capture the intact structures of MoS_2 (blue) and V-MoS_2 (pink) monolayers, respectively, without radiation-induced structural damage. Scale bars in the ADF images are 2 nm.

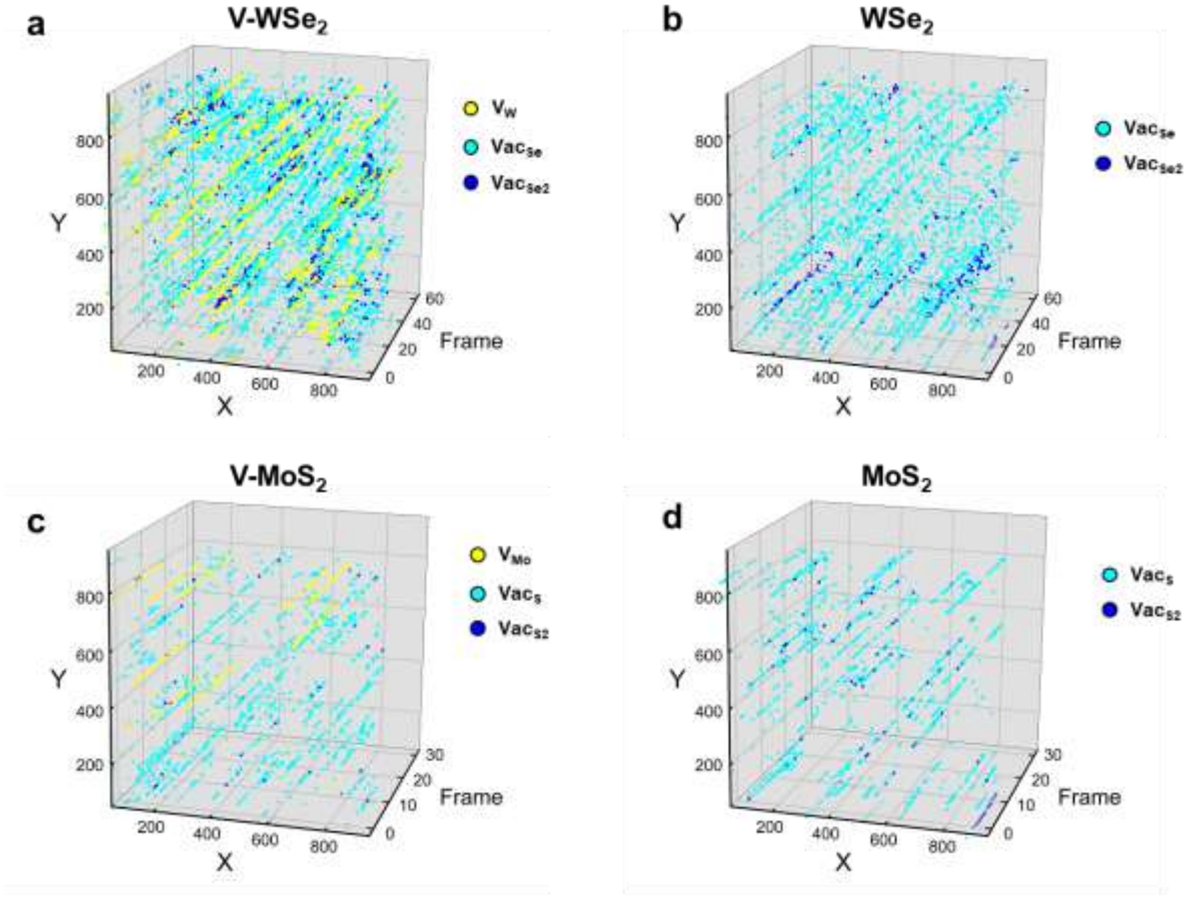

Figure S8. Spatiotemporal trajectory analysis of the V dopants and chalcogen vacancies for a) V-WSe_2, b) WSe_2, c) V-MoS_2, and d) MoS_2 samples, respectively. These 3D trajectory diagrams describe how the defects evolve and transition under electron beam irradiation, which can be readily visualized from the results of stacked site classification maps obtained by the automated quantification algorithm.
